# Development and Validation of a Tobacco Testing Laboratories Assessment Tool

**DOI:** 10.1371/journal.pone.0334653

**Published:** 2025-11-07

**Authors:** Sonu Goel, Chirag Goel, Diksha Walia, Avinash Sunthlia, Leimapokpam Swasticharan, Rajendra P. Joshi, Atul Goel

**Affiliations:** 1 Public Health Master’s Program, School of Medicine, University of Limerick, Limerick, Ireland; 2 Department of Community Medicine and School of Public Health, Post Graduate Institute of Medical Education and Research (PGIMER), Chandigarh, India; 3 Faculty of Human and Health Sciences, Swansea University, Swansea, United Kingdom; 4 Deputy Additional Director General- NTCP, Ministry of Health and Family Welfare, Government of India, New Delhi, India; 5 Additional Deputy Director General of Health Services, Directorate General of Health Services, Ministry of Health and Family Welfare, Government of India, New Delhi, India; 6 Additional Directorate General of Health Services, Ministry of Health and Family Welfare, Government of India, New Delhi, India; 7 DGHS, Directorate General of Health Services, Ministry of Health and Family Welfare, Government of India, New Delhi, India; AIIMS Jodhpur: All India Institute of Medical Sciences - Jodhpur, INDIA

## Abstract

**Background:**

Effective regulation and control of tobacco products require robust laboratory testing capabilities to ensure quality, safety and compliance with relevant standards. Currently, no standardized assessment tool exists globally to evaluate the tobacco testing laboratories. This study aims to address this gap by developing the Tobacco Testing Laboratory Assessment Tool (TTLAT). This tool aligns with the WHO-FCTC on Tobacco Control’s Article 9, which calls for the adoption and implementation of effective testing, measuring, and regulation measures.

**Methodology:**

The TTLAT was developed through a systematic literature review and a two-round Delphi technique involving 24 experts. The tool was then validated in four National Tobacco Testing Laboratories in India. Content validity was assessed using the Item-Content Validity Index (I-CVI) and Scale-Content Validity Index (S-CVI). Construct validity was evaluated through exploratory and confirmatory factor analyses. Internal consistency reliability was measured using Cronbach’s alpha.

**Results:**

The final TTLAT comprises 213 items across 11 critical domains including General Information (17), Documents (37), Organization and Management (10), Human Resources (28), Infrastructure (7), Equipment (23), Consumables and Reagents (10), Sample Handling (17), Tobacco Product Analytes (8), Data Management (36), and Biosafety and Biosecurity (20). Content validity analysis showed excellent results. Exploratory factor analysis identified six factors accounting for 52.5% of the total variance. Confirmatory factor analysis demonstrated good model fit (CFI = 0.91; TLI = 0.90; RMSEA = 0.05; SRMR = 0.06). The tool showed high internal consistency reliability across factors (Cronbach’s alpha 0.72–0.92).

**Conclusion:**

The TTLAT demonstrates strong psychometric properties and provides a comprehensive, standardised approach for assessing tobacco testing laboratory capacity.

## 1. Introduction

Tobacco use, encompassing traditional forms such as cigarettes and cigars and newer alternatives like electronic nicotine delivery systems (ENDS), electronic non- nicotine delivery systems (ENNDS) and heat-not-burn (HNB) devices, is a major global health crisis [1,2] causing an estimated 8 million deaths annually [3]. The harmful chemicals in these products, including nicotine, tar, volatile organic compounds (VOCs), heavy metals and carcinogens, contribute to various cancers, cardiovascular diseases, and respiratory illnesses [4–6].

To overcome the growing threat of tobacco use, the World Health Organization (WHO) negotiated the Framework Convention on Tobacco Control (FCTC) in 2003 [7]. It represents the first-ever global public health effort solely dedicated to tobacco control with an objective to address the widespread tobacco epidemic worldwide and safeguard current and future generations from the severe health, social, environmental, and economic repercussions of tobacco use and exposure to tobacco smoke. Among its key provisions are Articles 9 and 10, which regulate tobacco product contents and emissions [7]. Under Article 9 of FCTC, the parties, in consultation with competent international bodies, should propose guidelines for testing and measuring the contents and emissions of tobacco products so that they can be adopted and implemented by the signatories. Each party, where the national authority approves, should adopt and implement effective testing, measuring, and regulation measures [7]. Under Article 10, manufacturers and importers need to disclose information about the contents and emissions of tobacco products besides indicating their toxic constituents to the public [7].

Effective regulation and control of tobacco products require robust laboratory testing capabilities to test different tobacco product analytes utilizing standard methodologies, biosafety procedures, and quality assurance and quality control methods. Several countries have established testing infrastructures to comply with Articles 9 and 10 of the WHO FCTC. Brazil has designated the National Institute of Metrology, Quality and Technology (Inmetro) as its tobacco testing laboratory, which measures tar, nicotine, carbon monoxide, and other emissions [8]. Under Health Canada, Canada’s Tobacco Control Programme Laboratory conducts similar tests and discloses results publicly [9]. The European Union has harmonized testing protocols across designated labs in each member state [10]. In the U.S., the Centers for Disease Control and Prevention (CDC) tests tobacco products [11], while the Food and Drug Administration (FDA) requires manufacturers to report harmful constituents [12]. Assessing the capacity of these laboratories is an essential first step in identifying areas for improvement and guiding capacity-building efforts to ensure product safety and compliance with relevant standards.

Research into the implementation of the WHO Framework Convention on Tobacco Control (FCTC) has highlighted significant gaps in laboratory capacity for testing and regulating tobacco products across many countries [13]. While several laboratory assessment tools have been developed viz. Laboratory Assessment Tool for laboratories implementing SARS-CoV-2 testing [14], the Assessment tool for laboratories implementing COVID-19 virus testing [15] Interim and the Laboratory Quality Management System [16] however they are not fully equipped to address the unique challenges faced by tobacco testing laboratories [13,17]. Tobacco testing laboratories often require specialized equipment, testing methodologies, and safety protocols that are not adequately covered by more generalized assessment frameworks. Therefore, developing assessment tools specifically for tobacco testing laboratories would be valuable for identifying areas needing improvement and guiding capacity-building initiatives to ensure product safety and compliance with relevant standards.

India has also taken steps to implement Articles 9 and 10 of the WHO Framework Convention on Tobacco Control (FCTC) through its domestic legislation, the Cigarettes and Other Tobacco Products (Prohibition of Advertisement and Regulation of Trade and Commerce, Production, Supply and Distribution) Act, 2003 (COTPA, 2003) [18]. In 2019, the Indian government formally acknowledged three National Tobacco Testing Laboratories (NTTLs) through a Gazette Notification [19]; these laboratories can also serve as regional references and testing laboratories for all sorts of tobacco products. This recognition, granted under the authority of section 11 of the COTPA Act, 2003 [20], empowers these labs to test the nicotine and tar levels in cigarettes and various other tobacco products.

This study aims to establish a comprehensive, standardized assessment tool—the Tobacco Testing Laboratory Assessment Tool—to evaluate the capacity and quality of tobacco testing laboratories. Currently, no such tool exists globally. Developing the TTLAT is crucial for enhancing tobacco product testing and regulation worldwide, especially given the evolving landscape of tobacco products and the need for robust testing methodologies [13]. This initiative aligns with the WHO FCTC’s Article 9, which calls for parties to adopt and implement effective testing, measuring, and regulation measures. The TTLAT will help laboratories meet international standards and best practices, ultimately contributing to more effective tobacco control efforts.

## 2. Methodology

**Fig 1:**
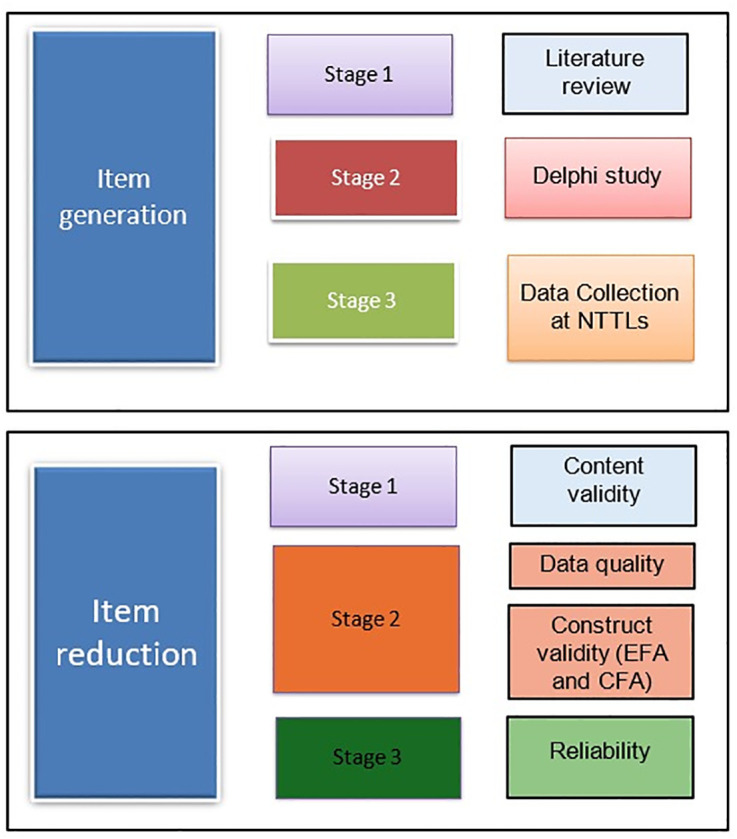
Steps for development and validation of the Tobacco Testing Laboratory Assessment Tool (TTLAT).

The Tobacco Testing Laboratory Assessment Tool (TTLAT) was developed through two major phases, namely: (1) Development of TTLAT (item generation) and (2) Validation of TTLAT (Item reduction) (Fig 1).

### 2.1. Item generation

#### 2.1.1. Literature review.

The study team conducted a systematic literature review to identify existing approaches and tools for assessing laboratory capacity, particularly in the context of tobacco testing. The review involved searching electronic bibliographic databases such as PubMed, Scopus, and Web of Science using a combination of keywords related to tobacco, laboratory assessment, and capacity building. In addition, the team reviewed relevant grey literature, including reports, guidelines, and assessment tools from international organizations such as the World Health Organization (WHO), Centres for Disease Control and Prevention (CDC), and International Organization for Standardization (ISO) [21–23]. The review yielded a set of key domains and components that are critical for the assessment of tobacco testing laboratories. The search strategies and results are described in S1 Table.

The literature review yielded a large number of relevant articles, which were then screened and selected based on predefined inclusion and exclusion criteria. The inclusion criteria for studies were: 1) Original articles and tools on assessing laboratory capacity, particularly in the context of tobacco testing, and relevant grey literature (reports, guidelines, and assessment tools from international organisations such as the World Health Organisation, Centres for Disease Control and Prevention, and International Organisation for Standardisation) 2) studies that were published in English. The Exclusion criteria were: 1) Studies or tools not mainly focused on laboratory assessment or building capacity 2) Studies or tools not relevant to tobacco testing/analytical laboratories, 3) Non-English language articles due to reasons of feasibility 4) Studies without full-text availability 5) studies not presenting novel data (opinion pieces, conference abstracts, commentaries, consensus statements, *in vitro* studies or letters to the editor)

#### 2.1.2. Tool development.

Delphi’s technique followed the literature review, in which a group of experts reached to a consensus on the domains/items in the tool through structured communication [24]. Two rounds of Delphi were conducted. In the first round conducted during the third week of January 2024, the key domains (S2 Table) and components of the proposed tool, which were extracted from the literature review were presented to a panel of 24 experienced experts, including senior laboratory scientists (n = 20) and public health consultants (n = 4), from various research institutes of international and national eminence. The majority of the experts had a Ph.D. in laboratory sciences, and more than 66% had at least 8 years of work experience in the field.

In the initial round, preliminary domains were established, and questions specific to smoke and smokeless tobacco products were proposed for the tobacco products analytes domain. Additional features, including a GAP Analysis section and equipment inventory tables, were also suggested. Two weeks later, during the second round, a panel of 24 experts was asked to rate their agreement with each domain on a 4-point scale (Not relevant, somewhat relevant, quite relevant, very relevant), and evaluate potential additional features for the assessment tool. Consensus was defined as 75% or higher agreement among panellists. The percentage agreement was calculated for each domain of the scale, allowing for iterative refinement of the assessment tool based on expert feedback.

### 2.2. Tool validation

#### 2.2.1. Study setting and data collection.

The data collection was conducted during March 2024 at four National Tobacco Testing Laboratories (NTTL) in India – NTTL- Noida, NTTL- Mumbai, the NTTL- Guwahati, and NIMHANS in Bangalore. These laboratories are established by the Ministry of Health and Family Welfare, Government of India, to provide scientific and analytical information to the Government of India and other regional countries and organisations such as the World Health Organization. Two trained researchers with prior experience in laboratory research collected data through semi-structured interviews with laboratory directors, scientists and technical assistants, as well as by reviewing relevant documents and observing laboratory operations.

#### 2.2.2. Content validity.

The content validity of the Tobacco Testing Laboratory Capacity Assessment Tool was evaluated by a panel of 24 senior scientists and academicians (different from the original panel included in the Delphi exercise) with extensive experience in the field of tobacco control and laboratory testing. The experts were asked to rate each item on a 4-point scale for its relevance, a 3-point scale for clarity, and a 3-point scale for essentiality. 1. CVI: The content validity index (CVI) is calculated using the Item-CVI (I-CVI) and Scale-level-CVI (S-CVI) [25]. I-CVI is computed as the number of experts rating an item as “very relevant” divided by the total number of experts. Items with I-CVI > 0.79 are considered relevant, while those between 0.70–0.79 need revisions, and those below 0.70 are eliminated [25]. The S-CVI can be calculated using two methods: the Universal Agreement (S-CVI/UA) and the Average CVI (S-CVI/Ave), with the latter being a less conservative approach. A S-CVI/UA ≥ 0.8 and a S- CVI/Ave ≥ 0.9 indicate excellent content validity [26].

2. CVR: CVR varies between 1 and −1, and a higher score indicates greater agreement among panel members regarding the essentiality of an item. The formula for calculating CVR is CVR = (Ne - N/2)/ (N/2), where Ne is the number of panellists indicating an item as “essential” and N is the total number of panellists [25].

#### 2.2.3. Construct validity and reliability.

Exploratory factor analysis (EFA) with varimax rotation was used to measure the construct validity of the tool. Cronbach’s alpha was calculated to assess the internal consistency of the subscales and the overall TTLAT [27]. Items showing a high correlation (r ≥ 0.90) or low correlation (r ≤ 0.30) with other items were dropped from the analysis. The Kaiser-Meyer-Olkin (KMO) test was used to assess the sampling adequacy, which should be > 0.5 for a satisfactory factor analysis to proceed [28,29]. Bartlett’s test was applied to check the strength of the relationships among the items. The criterion of an Eigenvalue ≥ 1 was used to define the number of factors to be retained. Items were loaded into factors based on factor loadings > 0.5 and < 0.4 to the rest of the factors [30].

Confirmatory factor analysis was then performed on the factors identified in the EFA to confirm the factor structure and assess the validity and reliability of the latent constructs [31,32]. The CFA was conducted using structural equation modelling (SEM) techniques [33]. The fit of the CFA model was evaluated using several goodness-of-fit indices, including the chi-square test (χ2), the comparative fit index (CFI), the Tucker-Lewis index (TLI), the root mean square error of approximation (RMSEA), and the standardized root mean square residual (SRMR) [34].

All data analyses were performed using the Statistical Package for the Social Sciences (SPSS IBM, New York, NY) and SPSS/Amos software (Version 21).

### 2.3. Ethical approval

Ethical approval for the study was obtained from the Institutional Ethics Committee at the Post Graduate Institute of Medical Education and Research (PGIMER) IEC-04/2021–1966, Chandigarh. Informed consent was obtained from all participants before data collection.

## 3. Results

### 3.1. Tool development

The literature review identified 230 items under 11 domains (S2 Table) that are critical for the assessment of tobacco testing laboratories, including 1) General Information (17), 2) Documents (37), 3) Organization and Management (10), 4) Human Resources (28), 5) Infrastructure (7), 6) Equipment (23), 7) Consumables and Reagents (10), 8) Sample Handling (17), 9) Tobacco Product Analytes (8), 10) Data Management (36), and 11) Biosafety and Biosecurity (20).

The Delphi technique involving 24 experts (20 laboratory experts and 4 public health consultants) was used to achieve consensus on the items in the tool. This method yielded significant consensus among the participants. The second Delphi round achieved high consensus across all refined domains, with agreement percentages ranging from 85% to 98%. The “General Information” domain received the highest consensus (98%), while “Biosafety and Biosecurity” received the lowest, though still high, consensus (85%). Significant changes were made to several domains based on expert feedback (Table 1). The proposed 1–4 scoring system (Not relevant, somewhat relevant, quite relevant, very relevant) received very high consensus (95%). Additional features such as a GAP Analysis section (80% consensus) and Equipment inventory tables (75% consensus) also met the threshold for inclusion. The final version of the tool comprised 213 unique questions organised into 11 domains (S2 Table). Overall, the iterative process resulted in a comprehensive tool structure with high expert consensus. The questionnaire development process is represented in Fig 1.

**Table 1:** Delphi result.

Refined Domains (Delphi Round 1)	Delphi Round II	
	Consensus % (n = 24)	Changes made
General Information	98%	Added GPS coordinates
Documentary Records	95%	Added a question about how long documentary records should be kept
Organisation and Management	90%	Added accreditation details
Human Resources	95%	Included continuous education
Infrastructure	90%	Added specific area requirements
Equipment	95%	Included detailed inventory table
Consumables and Reagents	90%	Added expiry management
Sample Collection, Handling and Transport	92%	Expanded from initial “Sample Handling” to “sample collection, handling and transport”
Tobacco Product analytes	88%	The question specific to smoke/smokeless products to be added
Data and Information Management	93%	Expanded from initial “Data Management” to “Data and Information Management”
Biosafety and Biosecurity	85%	Added disposal of tobacco product samples
**Final Scoring System**		
1–4 scale (Yes, Partial, No, N/A)	95%	
**Additional Features**		
GAP Analysis section	80%	
Equipment inventory tables	75%	

### 3.2. Tool validation

#### 3.2.1. Content validity.

The content validity analysis showed excellent results. The expert panel’s evaluation of each item’s relevance, clarity, and essentiality also supported the tool’s content validity. The majority of items were rated as “quite relevant” or “very relevant,” “item needs some revision,” “very clear,” and “useful, but not essential” or “essential.”

#### 3.2.2. I-CVI results (relevancy of individual items).

198 items (93%) were marked as relevant, and the Item-CVI (I-CVI) ranged from 0.5 to 1.00. The majority of the items were considered relevant (having an I-CVI greater than 0.79), except for 13 items: five in documentary records, four in organization and management, one in quality official, and three in the equipment section.

#### 3.2.3. S-CVI results (relevancy of overall tool).

The Scale-level-CVI/Universal Agreement (S-CVI/UA) was 0.84, and the Scale- level-CVI/Average (S-CVI/Ave) was 0.93, both of which exceed the recommended thresholds of 0.8 and 0.9, respectively, for excellent content validity [35,36].

#### 3.2.4. CVR results (essentiality).

The content validity ratio (CVR) ranged from 0.60 to 1.00, with all items having a CVR greater than the critical value of 0.37 for a panel size of 24 [37]. This indicates that all items were considered essential by the expert panel.

#### 3.2.5. Clarity results (individual items and overall tool).

The average clarity scores of the TTLCAT on a 3-point Likert scale for individual items ranged from 2.35 to 3.00, with 30 items (14%) regarded as very clear, while 32 items (15%) had an average clarity score of 3.00, 65 items (31%) have a score of 2.85, 68 items (32%) a score of 2.68, and 18 items (8%) a score of 2.35. The overall clarity score of the tool was 2.78.

### 3.3. Construct validity and reliability

The exploratory factor analysis (EFA) identified 6 factors that accounted for 52.5% of the total variance [38,39]. The Kaiser-Meyer-Olkin (KMO) measure of sampling adequacy was 0.88, and Bartlett’s test of sphericity was significant (p < 0.001), indicating that the data were suitable for factor analysis.

**Fig 2:**
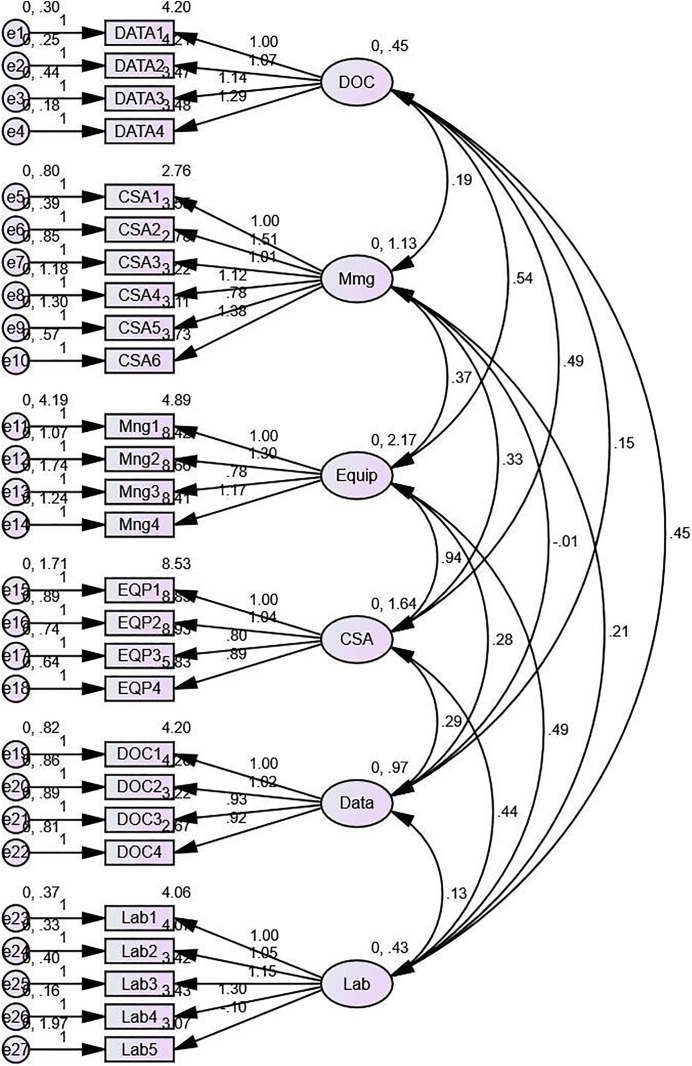
Confirmatory Factor Analysis Model of the Tobacco Testing Laboratory Assessment Tool (TTLAT). This figure illustrates the structural equation model resulting from the confirmatory factor analysis (CFA) of the TTLAT. Rectangles: Represent observed variables (individual items) from the TTLAT. Ovals: Represent latent constructs (factors) identified in the exploratory factor analysis. Single-headed arrows: Indicate factor loadings, showing the relationship between latent constructs and their respective observed variables. Double-headed curved arrows: Represent correlations between latent constructs. Numbers on arrows: Standardised factor loadings or correlation coefficients. F1-F6: Labels for the six factors identified: F1: General Information and Documentation F2: Organization and Management F3: Infrastructure and Equipment F4: Consumables and Tobacco Sample Testing F5: Data and Information Management F6: Biosafety and Biosecurity.

The confirmatory factor analysis (CFA) was performed on the six factors identified in the EFA (Fig 2). The CFA model demonstrated good fit indices (GFI): 0.870, p < 0.001; CFI = 0.91; TLI = 0.90; RMSEA = 0.05 (90% CI: 0.047–0.053); SRMR = 0.06. All factor loadings were statistically significant (p < 0.001), and the standardised factor loadings ranged from 0.55 to 0.89, indicating that the items were well represented by their respective factors.

The internal consistency reliability of the six factors was assessed using Cronbach’s alpha. All factors showed acceptable reliability, with Cronbach’s alpha values ranging from 0.72 to 0.92, demonstrating excellent internal consistency [38,40].

The six factors identified through the EFA and CFA were:

1. General Information and Documentation (α = 0.92): This domain covers the basic information about the tobacco testing laboratory, such as its name, location, contact details, and any relevant accreditations or certifications. It also examines the availability and organisation of the laboratory’s documentation, including standard operating procedures, testing protocols, equipment manuals, and quality management records. This domain ensures that the laboratory has the necessary foundational information and documentation to support its operations and demonstrate its competence.
2. Organisation and Management (α = 0.90): This domain examines the organisational structure, leadership, and governance of the laboratory, as well as its legal and regulatory framework.
3. Infrastructure and Equipment (α = 0.87): This domain evaluates the physical infrastructure, equipment, and facilities available for tobacco testing
4. Consumables and Tobacco Sample testing (α = 0.79): This domain examines the laboratory’s operational processes, including sample handling, testing methodologies, and quality assurance/quality control measures
5. Data and Information Management (α = 0.82): This domain assesses the laboratory’s data management systems, data security, and reporting processes.
6. Biosafety and Biosecurity (α = 0.72): This domain examines the laboratory’s policies, procedures, and practices to ensure the safe handling of tobacco samples and protect personnel and the environment from potential hazards. This includes the implementation of appropriate biosafety measures, such as personal protective equipment, containment facilities, waste disposal, and decontamination protocols. The domain also evaluates the laboratory’s biosecurity measures to prevent the unauthorised access, loss, theft, or misuse of tobacco samples and related materials.

## 4. Discussion

A comprehensive Tobacco Testing Laboratory Assessment Tool was developed and validated to evaluate tobacco product testing laboratories. The rigorous development process involved a literature review, Delphi technique, and field testing in four major Indian national tobacco testing laboratories. The final tool includes 213 items across 11 domains, aligning with international standards and best practices [7,41]. The Delphi technique played a key role in refining the TTLAT. This iterative consensus- building method engaged 24 experts, including laboratory experts and public health consultants, enabling the incorporation of diverse perspectives [42,43]. The high levels of agreement achieved in the second round, particularly for core sections like General Information (98%) and Equipment (95%), underscore the tool’s comprehensive coverage of critical laboratory aspects. The refinements made to each domain reflect a thorough consideration of practical and regulatory aspects of tobacco product testing laboratories. The expansion of “Sample Handling” to include collection and transport acknowledges the importance of pre-analytical factors in ensuring reliable results. Similarly, the broadening of “Data Management” to “Data and Information Management” recognises the complex informational ecosystems in modern laboratories. The addition of questions about record retention periods in the “Documentary Records” domain highlights the importance of proper documentation practices in regulatory compliance and quality assurance. This change aligns with expert recommendations on maintaining traceable and auditable records in scientific laboratories. The addition of questions specific to Smoke and Smokeless Tobacco Products (both at 88% agreement) reflects the diverse nature of tobacco products and the need for specialised assessment criteria. This differentiation enhances the tool’s applicability across diverse tobacco product testing. The near-unanimous approval of the 1–4 scale scoring system suggests its perceived effectiveness in capturing the nuanced realities of laboratory capabilities. The inclusion of a GAP Analysis section (80% agreement) demonstrates a forward-thinking approach, allowing laboratories to identify areas for improvement. Similarly, the equipment inventory tables (75% agreement) provide a practical means of assessing technical capabilities. These features transform the tool from a mere assessment instrument to a catalyst for strengthening the laboratory.

The Delphi technique proved particularly well-suited for tool development in specialised fields like tobacco testing, where expert judgment is crucial. Compared to other Delphi-based tool development studies, this research stands out in the rapid achievement of consensus and the retention of all initially identified items, suggesting a high level of initial agreement among experts. This may be attributed to the highly specialised nature of tobacco testing and the comprehensive initial literature review [44,45]. Similar Delphi-based tool development studies in environmental health and food safety have combined literature reviews with expert input, as with this approach used in the current study [46,47]. However, these often-involved smaller expert panels or used quantitative scoring systems, unlike the more qualitative approach employed in this study. Additionally, the inclusion of both laboratory experts and public health consultants on the panel provides a balanced perspective, an approach not always seen in other Delphi-based tool development studies.

While the Delphi method offers advantages in tool development, assessing the validity of the resulting tool presents its challenges. Our study considered both Content and construct validity to reach out to the overall validity of the tool. The most widely used method for assessing content validity is the Item-level Content Validity Index (I-CVI) [35,39]. However, an alternative, lesser-known approach is the Scale-level CVI (S-CVI), which can be calculated using either the S- CVI/Universal Agreement (UA) or the S-CVI/Average (Ave) method [25,48]. These two approaches may yield different values, making it challenging to draw definitive conclusions about content validity [39]. While the I-CVI evaluates the content validity of individual items, the S-CVI assesses the content validity of the overall scale [35,48]. Most research papers report either the I-CVI or the S-CVI, but not both [48]. This study considered both indices since the S-CVI, being an average score, can be skewed by outliers. The number of experts (n = 24) was deemed adequate for content validation, as the recommended range is between 3 and 10 raters [36,49]. An I-CVI of 0.78 or higher is considered excellent [50]. The I-CVIs of all items in the tool ranged from 0.50 to 1.00, with only 13 items having an I-CVI below 0.79. This supports the conclusion that individual items were important and relevant for measuring the capacity of tobacco testing laboratories. The minimum acceptable S-CVI is typically between 0.80 and 0.90 [36,39]. The Universal Agreement approach suggested good overall content validity for the tool (S-CVI/UA = 0.84), while the Average method indicated high content validity (S-CVI/Ave = 0.93). Although the Universal Agreement method, which only considers items with an I- CVI of 1.00, may be regarded as more comprehensive, it may underestimate the overall content validity as the likelihood of achieving 100% agreement decreases with an increasing number of experts [51]. Conversely, the less restrictive S- CVI/Ave approach may overestimate content validity since the numerator will always be greater than the Universal Agreement approach if I-CVI values are not equal to 1.00 [48]. For this reason, both the S-CVI/UA and the S-CVI/Ave were calculated, and the true overall content validity of the tool may lie somewhere in between. A less common method for calculating content validity is the Content Validity Ratio (CVR) approach, which determines how many raters consider an item essential. The content validity ratio (CVR) further supported the essentiality of the items, with all items exceeding the critical value [37].

Furthermore, the construct validity of the tool was established through exploratory and confirmatory factor analyses [52,53]. The EFA identified six factors that accounted for a substantial portion of the total variance (52.5%), and the CFA confirmed the factor structure, demonstrating good model fit and supporting the validity of the latent constructs [54,55]. This robust statistical analysis supports the tool’s ability to measure the intended constructs accurately [56]. The high internal consistency reliability of the factors, as indicated by Cronbach’s alpha values, further reinforces the reliability of the tool in assessing the capacity of tobacco testing laboratories [57,58].

Overall, the comprehensive validation process and the strong psychometric properties of the TTLAT affirm its reliability and validity as a tool for assessing the capacity of tobacco testing laboratories, which shall potentially enhance the quality and standardisation of laboratory testing practices in the field of tobacco control. Previous laboratory assessment tools have focused on general laboratory capacity or specific disease areas, but none have been tailored to tobacco testing laboratories’ unique needs and requirements [13,17]. By providing a comprehensive framework for assessing critical domains such as organisational structure, human resources, infrastructure, operational processes, data management, and sustainability, the tool offers a standardised approach for evaluating laboratory capacity. This can lead to improved consistency and reliability in tobacco testing procedures, ensuring the accuracy and integrity of test results. Furthermore, the TTLAT holds promise in capacity-building efforts, informing policy and decision-making related to tobacco testing and regulation. Its ability to capture essential aspects of laboratory performance and capacity can guide policymakers in implementing quality standards, resource allocation, and regulatory measures to ensure the effectiveness and credibility of tobacco testing practices.

Additionally, the TTLAT’s reliability and validity make it a valuable resource for fostering continuous improvement and accreditation efforts within tobacco testing laboratories. The tool acts as a gap analysis instrument. It helps laboratories to find areas for improvement before engaging with accreditation bodies. This can lead to shorter timelines and lower costs [59]. The inclusion of tobacco-specific requirements within broader laboratory quality frameworks makes the TTLAT useful for tobacco testing facilities. It connects general laboratory standards with tobacco-specific regulations. [60].

The TTLAT also supports the global implementation of WHO FCTC Articles 9 and 10. It tackles important gaps in international tobacco testing infrastructure [7]. By setting standardised assessment criteria, the TTLAT helps countries measure their laboratory capabilities against global best practices. This promotes progress toward FCTC compliance among 182 parties [61]. The tool focuses on testing methods and quality assurance, which supports the scientific standards needed for effective regulation of tobacco products, including new products like ENDS and heated tobacco products [62].

By identifying areas for enhancement and benchmarking against best practices, the tool can support laboratories in their pursuit of excellence, ultimately contributing to the advancement of the tobacco testing field. This is particularly important in low- and middle-income countries, where laboratory capacity for tobacco product testing is often limited [63].

## 5. Limitations

The study faced several challenges, particularly in the expert consultation phase. Firstly, the diversity of individual expertise among the subject matter experts (SMEs) posed challenges when relying on team consensus for the overall Tool development. Second, it became evident that a more targeted approach to engaging SMEs, leveraging their specific skills (e.g., having tobacco laboratory SMEs review only the tobacco analytes sections), could have been more efficient and potentially reduced the time required to reach a consensus. Third, while the input from a diverse group of SMEs ultimately contributed to creating a Tool tailored to the specific needs and goals of the project, this approach may have introduced some inconsistencies across different sections of the Tool.

## 6. Conclusion

In conclusion, the rigorous validation process and the compelling psychometric properties of the TTLAT position it as a pivotal tool for assessing the capacity of tobacco testing laboratories. Its comprehensive coverage of critical domains, strong content and construct validity, and high internal consistency underscore its potential to drive quality improvement, guide capacity-building efforts, inform policy decisions, and facilitate accreditation in the realm of tobacco testing. As the field of tobacco control continues to evolve, the TTLAT stands as a foundational asset for ensuring the robustness and reliability of laboratory testing practices. It can be adapted to other similar geographical locations and laboratory facilities. Future research should aim to conduct an analysis on laboratory capacity research and identify areas for targeted intervention between developed and developing countries to better understand the global disparities.

## Supporting information

S1 Table Number of results obtained from each search strategy in the respective database. (DOCX)

S2 Table Assessment domains for tobacco testing laboratories. (DOCX)
