# Supplementary material for: Development and Validation of a Tobacco Testing Laboratories Assessment Tool
Source: PLoS One. 2025 Nov 7;20(11):e0334653. doi: 10.1371/journal.pone.0334653 (PMC12594407; doi:10.1371/journal.pone.0334653)
Supplement: S1 Table — (DOCX) [file pone.0334653.s001.docx]

SUPPLEMENT

**Table 1:** Number of results obtained from each search strategy in the respective database.

| 1. Database: PubMed. | | |
| --- | --- | --- |
| **Concept** | **Search strategy** | **Hits** |
| Tobacco laboratory testing | (Tobacco[Title/Abstract]) AND (("Laboratory assessment"[Title/Abstract] OR "Laboratory evaluation"[Title/Abstract] OR "Laboratory quality control"[Title/Abstract] OR "Test validation"[Title/Abstract] OR "Laboratory proficiency test*"[Title/Abstract] OR "Quality assurance"[Title/Abstract] OR "Laboratory documentation"[Title/Abstract] OR "Laboratory organization"[Title/Abstract] OR "Laboratory management"[Title/Abstract] OR "Laboratory infrastructure"[Title/Abstract] OR "Laboratory biosafety"[Title/Abstract] OR "Laboratory equipment"[Title/Abstract] OR "Laboratory biosafety"[Title/Abstract] OR "Laboratory biosecurity"[Title/Abstract]) OR (("Laboratory assessment tool"[Title/Abstract] OR ("Laboratory test*"[Title/Abstract]) OR "Laboratory accreditation"[Title/Abstract] OR "Laboratory quality control"[Title/Abstract] OR National accreditation  board for testing and calibration laboratories[Title/Abstract] OR "NABL"[Title/Abstract]) OR (test*[Title/Abstract]))) | 17,017 |
| Tobacco laboratory testing in India | ((tobacco[Title/Abstract]) AND (("Laboratory assessment"[Title/Abstract] OR "Laboratory evaluation"[Title/Abstract] OR "Laboratory quality control"[Title/Abstract] OR "Test validation"[Title/Abstract] OR "Laboratory proficiency test*"[Title/Abstract] OR "Quality assurance"[Title/Abstract] OR "Laboratory documentation"[Title/Abstract] OR "Laboratory organization"[Title/Abstract] OR "Laboratory management"[Title/Abstract] OR "Laboratory infrastructure"[Title/Abstract] OR "Laboratory biosafety"[Title/Abstract] OR "Laboratory equipment"[Title/Abstract] OR "Laboratory biosafety"[Title/Abstract] OR "Laboratory biosecurity"[Title/Abstract]) OR (("Laboratory assessment tool"[Title/Abstract] OR ("Laboratory test*"[Title/Abstract]) OR "Laboratory accreditation"[Title/Abstract] OR "Laboratory quality control"[Title/Abstract] OR National accreditation board for testing and calibration laboratories[Title/Abstract] OR  "NABL"[Title/Abstract]) OR (test*[Title/Abstract])))) AND (India[Title/Abstract]) Sort by: Most Recent | 505 |

| 1. Database: Scopus. | | |
| --- | --- | --- |
| **Concept** | **Search strategy** | **Hits** |
| Tobacco laboratory testing | ( TITLE-ABS ( tobacco ) AND TITLE-ABS ( India ) ) AND (  TITLE-ABS ( "laboratory assessment" OR "laboratory evaluation" OR "laboratory quality control" OR "test validation" OR "laboratory proficiency test*" OR "quality assurance" OR "laboratory document*" OR "laboratory organisation" OR "laboratory management" OR "laboratory infrastructure" OR "laboratory biosafety" OR "laboratory equipment" OR "laboratory biosafety" OR "laboratory biosecurity" OR "laboratory assessment tool" OR "laboratory test*" OR "laboratory accreditation" OR "laboratory quality control" OR "National Accreditation Board for Testing and Calibration Laboratories" ) ) | 8 |

| 1. Database: Web of Science. | | |
| --- | --- | --- |
| **Concept** | **Search strategy** | **Hits** |
| #1 India AND Tobacco | (TI=(tobacco)) OR AB=(tobacco) | 131,679 |
| #2 Tobacco laboratory testing | (TI=("Laboratory assessment" OR "Laboratory evaluation" OR "Laboratory quality control" OR "Test validation" OR "Laboratory proficiency test*" OR "Quality assurance" OR "Laboratory documentation" OR "Laboratory organisation" OR "Laboratory management" OR "Laboratory infrastructure" OR "Laboratory biosafety" OR "Laboratory equipment" OR "Laboratory biosafety" OR "Laboratory biosecurity" OR "Laboratory assessment tool" OR "Laboratory test*" OR "Laboratory accreditation" OR "Laboratory quality control" OR "National Accreditation Board for Testing and Calibration Laboratories" OR "NABL")) OR AB=("Laboratory assessment" OR "Laboratory evaluation" OR "Laboratory quality control" OR "Test validation" OR "Laboratory proficiency test*" OR "Quality assurance" OR "Laboratory documentation" OR "Laboratory organisation" OR "Laboratory management" OR "Laboratory infrastructure" OR "Laboratory biosafety" OR "Laboratory equipment" OR "Laboratory biosafety" OR "Laboratory biosecurity" OR "Laboratory assessment tool" OR "Laboratory test*" OR "Laboratory accreditation" OR  "Laboratory quality control" OR "National Accreditation Board for Testing and Calibration Laboratories" OR "NABL") | 142,470 |
| #1 AND #2 |  | 179 |
